# Cost-utility analysis of a collaborative and stepped care model in patients with mental disorders in German primary care (the COMET study)

**DOI:** 10.1186/s12888-025-07428-5

**Published:** 2025-10-13

**Authors:** Thomas Grochtdreis, Daniela Heddaeus, Tharanya Seeralan, Kerstin Maehder, Sarah Porzelt, Anne Daubmann, Amra Pepic, Bernd Löwe, Moritz Rosenkranz, Ingo Schäfer, Martin Scherer, Bernd Schulte, Olaf von dem Knesebeck, Angelika Weigel, Karl Wegscheider, Silke Werner, Antonia Zapf, Thomas Zimmermann, Jörg Dirmaier, Martin Härter, Hans-Helmut König, Judith Dams

**Affiliations:** 1 Department of Health Economics and Health Services Research, Hamburg Center for Health Economics, University Medical Center Hamburg-Eppendorf, Hamburg, Germany; 2 Department of Medical Psychology, University Medical Center Hamburg-Eppendorf, Hamburg, Germany; 3 Department of Psychosomatic Medicine and Psychotherapy, University Medical Center Hamburg-Eppendorf, Hamburg, Germany; 4 Department of General Practice and Primary Care, University Medical Center Hamburg- Eppendorf, Hamburg, Germany; 5 Department of Medical Biometry and Epidemiology, University Medical Center Hamburg-Eppendorf, Hamburg, Germany; 6 Center for Interdisciplinary Addiction Research, University of Hamburg; Department of Psychiatry and Psychotherapy, University Medical Center Hamburg-Eppendorf, Hamburg, Germany; 7 Department of Medical Sociology, University Medical Center Hamburg-Eppendorf, Hamburg, Germany; 8 Faculty of Economics and Social Sciences, Hamburg University of Applied Sciences, Hamburg, Germany

**Keywords:** Cost-effectiveness analysis, Depressive disorders, Anxiety disorders, Somatoform disorders, Alcohol-related disorders, Primary care, Collaborative care, Stepped care

## Abstract

**Background:**

In order to reduce the health burden and the health care costs caused by the most common mental disorders, health care systems throughout Europe have tried to improve services and treatment choices. Recently, a collaborative and stepped care (CSC) model for patients with depressive, anxiety, somatoform or alcohol-related disorders and their comorbidities was developed and implemented under routine care conditions in Germany. The aim of this study was to determine the cost-effectiveness of this CSC model from a societal perspective with a 12-month follow-up.

**Methods:**

This study was part of a cluster-randomized controlled trial to compare a CSC model with treatment as usual (TAU) in patients with depressive, anxiety, somatoform or alcohol-related disorders and their comorbidities in German routine care. The cost-effectiveness of the CSC model compared with TAU was analyzed based on the incremental cost-utility ratio (ICUR) with quality-adjusted life years (QALYs) based on the EQ-5D-5L index as measure of health effect. The uncertainty of the ICUR was assessed using cost-effectiveness acceptability curves based on net-benefit regressions.

**Results:**

In total, *n* = 307 patients in the CSC and *n* = 308 patients in the TAU group were included, with a mean age of 38 and 43 years, respectively. There were no differences in mean QALYs and total costs between the CSC (0.86 QALY, 27,174€) and the TAU group (0.86 QALY, 26,441€). Only the adjusted mean costs for outpatient mental health services were higher in the CSC group (+685€; 95% CI 398€ to 972€; *p* < 0.001). The probability of cost-effectiveness of the CSC model was 35% at a willingness-to-pay (WTP) of 0€ and 34% at a WTP of 50,000€ per additional QALY.

**Conclusion:**

The evaluated CSC model was unlikely to be cost-effective compared with TAU from a societal perspective for patients with depressive, anxiety, somatoform or alcohol-related disorders and their comorbidities during the 12-month follow-up period. The higher mean costs for outpatient mental health services might indicate that general practitioners in the CSC group were able to refer patients to psychotherapists and psychiatrists more frequently through the network of health care providers.

**Trial registration:**

ClinicalTrials.gov: NCT03226743. Registration date: 24/7/2017.

**Supplementary Information:**

The online version contains supplementary material available at 10.1186/s12888-025-07428-5.

## Introduction

Mental disorders highly contribute to the health burden of the European population [1]. The 12-month prevalence of the four most common mental disorders - depressive disorders, anxiety disorders, somatoform disorders and alcohol-related disorders - amounted up to 32% in the European Union in the year 2010. These mental disorders accounted for a large proportion of years of life lost due to premature mortality and years of life lost due to living with disability, resulting in more than seven million disability-adjusted life years caused by mental disorders in the European Union [1]. Furthermore, in 2010, these mental disorders caused annual costs of about 271 billion euros in the European Union, half of which were indirect costs resulting from absenteeism from work and overall reduced work productivity [2].

In order to reduce the health burden and health care costs caused by depressive disorders, anxiety disorders, somatoform disorders, and alcohol-related disorders, efforts have been made in recent years across Europe and especially in Germany to optimize health services and treatment (e.g [3–10]). In particular, collaborative care, i.e. the systematic integration of general practitioners (GPs), psychotherapists, psychiatrists, and other (mental) health specialists in evidence-based treatment, moved into the focus of efforts in this regard [11, 12]. As a complement to collaborative care, stepped care provides patients with mental disorders with treatment recommended in guidelines depending on the symptom severity of the underlying disorder and the response to treatment, and is often supported by a systematic monitoring of patient’s symptom severity [13]. In comparison, treatment as usual (TAU) under the German statutory health care system includes potential referrals to psychotherapy or psychiatric or psychosomatic outpatient or inpatient facilities by the GP [14]. Routine mental health care is provided on the basis of treatment recommendations that are based on research findings and clinical experience contained in clinical practice guidelines, yet neither systematic stepped care and symptoms monitoring nor a case management is regularly carried out.

The effectiveness of collaborative and stepped care (CSC) models was demonstrated in multiple systematic literature reviews for depressive [15, 16] and anxiety disorders [17], whereas for somatoform [18, 19] and alcohol-related disorders [20, 21] it was only examined in single studies. The cost-effectiveness of CSC models for depressive disorders was evaluated in three systematic literature reviews [22–24] and in several more recently published studies with a general focus on patients with depressive disorders [4, 6, 25], with a focus on elderly patients [7, 26–28], on pregnant women [29], and patients with somatic diseases and comorbid depression [30–33]. Overall, cost-effectiveness of CSC models for depressive disorders was ambiguous depending on the willingness-to-pay (WTP) for additional health effects [22]. For anxiety disorders, the cost-effectiveness of CSC models was evaluated in one systematic literature review [34] and in studies focusing on patients with anxiety disorders in general, and with panic disorder, generalized anxiety disorder and obsessive-compulsive disorder [6, 35–39], as well as on pediatric and youth patients [40, 41]. For somatoform disorders and alcohol-related disorders, the cost-effectiveness of CSC models was evaluated only rarely [5, 6, 20, 21].

The studies and the systematic literature reviews mentioned have in common that they mainly focus on one to two mental disorders and possibly comorbid disorders. Furthermore, CSC in these studies was often only partially implemented according to common definitions [11, 12, 22, 23]. In order to overcome those limitations, a CSC model for patients with the most common mental disorders, i.e. depressive disorders, anxiety disorders, somatoform disorders and alcohol-related disorders, was developed and implemented in the existing structures of primary care practices in Germany without having to resort to the support of additional care managers [14, 42]. Moreover, this model consisted of a cooperative multidisciplinary network of GPs, psychotherapists, psychiatrists, other mental health specialists as well as inpatient or day-care clinics, if necessary. It was guided by measures of symptom severity and provided patients with evidence-based treatment according to guideline-based stepped treatment recommendations using a tablet-based software.

The collaboration experiences of care providers and the effectiveness of this CSC model have already been evaluated [14, 43]. However, there is no evidence of cost-effectiveness of the CSC model so far. Therefore, the aim of this study was to evaluate the cost-effectiveness of the CSC model compared with TAU in patients with depressive, anxiety, somatoform or alcohol-related disorders and their comorbidities in German primary care from a societal perspective.

## Materials and methods

### Sample

This study was part of a prospective superiority trial with cluster-randomization and parallel groups of patients with mental disorders to compare the effectiveness of a CSC model with TAU in German primary care (the COMET study, trial registration: ClinicalTrials.gov, NCT03226743, registration date: 24/7/2017) [14, 42]. The primary endpoint of this trial was mental health-related quality of life (HrQoL) at 6 months after baseline. Primary care practices in the city of Hamburg were recruited by postal letters and phone contact, followed by an information event or a personal presentation of the study. The inclusion criterion of primary care practices was approval of the GP by the Hamburg Association of Statutory Health Insurance Physicians. In Germany, only approved GPs are allowed to run a local practice and to provide treatment to patients with statutory health insurance depending on the requirements planning of the local association of statutory health insurance physicians. After inclusion in the study, primary care practices were randomly assigned to the CSC or TAU group. The ratio of randomization was 1:1 and no stratification was used. Random numbers generated by a computer were used for randomization. Thereafter, each patient entering the primary care practice who gave written informed consent was screened for participation using a computer-assisted screening procedure. Due to the real-world setting of the study, randomization of patients could have led to CSC being inadvertently shared between groups and potentially distorted the results. Therefore, it was not possible to randomize patients rather than primary care practices and to blind participating primary care practices and patients to group assignment. The patient recruitment, intervention, and data assessment took place between July 2018 and October 2022. Patient inclusion criteria were being at least 18 years, sufficient knowledge of the German language, and written informed consent. The recruitment strategy had to be changed during the ongoing study, as some primary care practices had difficulty recruiting patients [14, 42]. In order to achieve the required sample size, one person from the study team supported recruitment at the medical practices concerned.

One or more of the following diagnoses from the International Statistical Classification of Diseases and Related Health Problems, 10th revision (ICD-10) from the block of affective disorders had to be determined by the GP: depressive episode, recurrent depressive disorder, and/or dysthymia. Furthermore, the following diagnoses from the block of neurotic, stress-related and somatoform disorders were available: agoraphobia, social phobia, panic disorder, generalized anxiety disorder, mixed anxiety and depressive disorder, and/or somatoform disorders. Furthermore, mental and behavioral disorders due to use of alcohol had to be determined by the GP for inclusion in the study. Patients were excluded if they were in current inpatient or outpatient psychotherapeutic care or psychopharmacotherapy.

Assessment of the study participants took place at baseline (T0), 6 months after baseline (T1) and 12 months after baseline (T2). A detailed description of the COMET study can be found elsewhere [14, 42].

### Interventions

Patients of the TAU group had unrestricted access to care as usual for the respective disorders. The GPs in the TAU group were instructed to continue current standard treatment. Patients of the CSC group participated in a collaborative and stepped care program provided by GPs, psychotherapists, psychiatrists, and inpatient or day-care clinics embedded in the standard health care system in Germany. The cooperating health care providers were integrated into a CSC network that met quarterly to improve the exchange of information on individual patient cases and on their work in general. Within the network, it was possible to book available treatment resources of psychotherapists, psychiatrists, and other mental health specialists for participating patients by GPs through an online scheduling platform provided by the Hamburg Association of Statutory Health Insurance Physicians. Furthermore, GPs were provided with a tablet-based software that gave one or more guideline-based treatment recommendations based on the severity of the diagnosed disorder of each patient participating in the study. Subsequently, GPs were encouraged to make an evidence-based treatment decision according to the principles of shared decision-making and patient-centered care [14, 42].

The CSC model consisted of eight possible interventions, which were structured in three steps with different intensity and setting. The first step included basic psychosocial care, psychoeducation, bibliotherapy, internet-based self-management, or single brief interventions (for alcohol-related disorders). The second step included psychotherapy or pharmacotherapy and the third step included psychotherapy plus pharmacotherapy or intensified treatment carried out by a multi-professional treatment team in a day-care clinic or inpatient facility. The process and outcome of treatment was monitored regularly by the GP or the mental health specialist, and treatment changes were made when necessary. Furthermore, a case management was implemented in order to point out opportunities for improvements to the GP in the treatment of patients with severe symptoms of the diagnosed disorder [14, 42].

The cooperating GPs and care providers of the CSC group received an initial training on the CSC model, four further trainings covering the guideline recommendations of the relevant disorders, and obtained detailed instruction manuals [14, 42].

### Measures

The generic HrQoL was measured using the five-level version of the EQ-5D (EQ-5D-5L) [44]. The EQ-5D-5L consists of five questions covering five dimensions of HrQoL: mobility, self-care, usual activities, pain/discomfort, and anxiety/depression. Each question is divided into five ordinal levels from ‘no problems’ to ‘extreme problems’. The descriptive system of the EQ-5D-5L is able to describe 3125 health states to which societal preferences can be assigned based on the German general population [45]. Based on these societal preferences for EQ-5D-5L health states, an EQ-5D-5L index can be calculated, that ranges from −0.661 (extreme problems in all 5 dimensions) to 1 (no problems in any dimension). In addition, subjective HrQoL was measured using the visual analogue scale of the EQ-5D-5L (EQ-VAS), and the SF-36 was used as alternative generic HrQoL measure [46, 47]. The EQ-VAS ranges from 0 (worst imaginable health state) to 100 (best imaginable health state) [44, 47]. Based on the SF-36, the mental component summary (MCS) score was calculated and SF-6D index scores were derived, which range from 0 (death) to 1 (full health) [46, 48].

Health-care service utilization was assessed using an adapted version of the German Client Socio-Demographic and Service Receipt Inventory [49]. The inventory covered questions on the utilization of inpatient care and rehabilitation, outpatient physician services, outpatient non-physician services, medical aids, medication, and formal and informal nursing care. Furthermore, patients were asked about their absenteeism and reduced productivity at work. Reduced productivity at work was assessed using a global rating scale ranging from 0 to 10 indicating overall work performance [50].

Socio-demographic characteristics were assessed by self-report of age, sex, marital status, migration background, education, occupational status, and type of health insurance. Furthermore, the main diagnosis and number of comorbidities were assessed by the GPs at baseline. The date of the baseline assessment was used to categorize the study inclusion of patients into before or after the onset of the COVID-19 pandemic. In addition, the study inclusion of patients was categorized into planned or changed framework of the recruitment strategy (with additional support from the study team), which became necessary to achieve the required sample size, especially in the TAU group, within the scheduled study period.

### Calculation of health effects

For use as health effect measure, quality-adjusted life years (QALYs) were calculated by weighting the time spent in the respective health state by the assigned EQ-5D-5L index score and summing the weighted scores [44, 45, 51]:

$$\:{QALY}_{T0-T2}=0.5\times\:\frac{{\textit{EQ-5D}}_\textit{T0}\mathit+{\textit{EQ-5D}}_\textit{T1}}2+\:0.5\times\:\frac{{\textit{EQ-5D}}_\textit{T1}\mathit+{\textit{EQ-5D}}_\textit{T2}}2$$

where T0, T1 and T2 are the assessment points and 0.5 adjusts for the duration between the assessments of the study participants (6 months = 0.5 years). Thereby, it was assumed that the HrQoL developed linearly between T0 and T1 and between T1 and T2, respectively. Negative EQ-5D-5L index scores were neither excluded nor adjusted and remained unchanged in the calculation of QALYs [52].

As secondary health effect measure, QALYs were calculated by dividing each EQ-VAS score by 100, weighting the time spent with the respective subjective HrQoL with those scores, and summing the weighted scores. Furthermore, QALYs were calculated based on SF-6D index scores in the same way as described above.

As additional secondary health effect measures, response to treatment and remission from symptoms were derived from symptom severity scores. As measures of symptom severity, the modules of the Patient Health Questionnaire [53] to screen for major depressive disorder (PHQ-9; range 0 to 27) [54], generalized anxiety disorder (GAD-7; range 0 to 21) [55] and somatoform disorders (PHQ-15; range 0 to 30) as well as the Alcohol Use Disorders Identification Test (AUDIT; range 0 to 40) [56] were used, with higher scores indicating greater severity. Response to treatment was defined as improvement of symptom severity scores by at least 50%. Remission from symptoms was defined as a PHQ-9 and GAD-7 score of < 5 and PHQ-15 score of < 9 [53, 57]. Furthermore, remission from symptoms was defined as an AUDIT score of < 4 and < 5 for females and males, respectively [56].

### Calculation of costs

Individual costs were calculated from a societal perspective in Euro (€) for the year 2019. Thereby, standardized unit costs for the German health care system were used to monetarily valuate the respective quantities of utilization of inpatient care and rehabilitation, outpatient physician services, outpatient non-physician services, medical aids and formal care [58–60]. The standardized unit costs were inflated to 2019 price levels based on the German consumer price index [61]. Costs of medication were calculated based on drug codes, and the number of medication packs used was monetarily valuated based on prices per pack [62]. Costs for informal care were calculated based on the replacement cost method. Thus, it was assumed that informal care could have been substituted by formal care, and therefore was monetarily valued based on the gross hourly wage of persons in the commercial sector ‘social care for older adults and disabled persons’ [58, 63]. The costs of absenteeism and reduced productivity at work were calculated based on the human capital approach. Thereby, absenteeism and reduced productivity at work were monetarily valuated based on the gross hourly wage of persons in manufacturing and services sectors [63]. For reduced productivity at work, one minus the overall work performance divided by 10 was multiplied by the days at work and the cost of one day completely absent from work.

Intervention costs consisted of costs for bibliotherapy and internet-based self-management, incentivization costs for the GP and mental health specialist attendance in the CSC network meetings, for the monitoring of the process and outcome of treatment, and for the use of the case management by GPs. Furthermore, intervention costs consisted of the labor costs for the participation to the CSC network meetings by GPs and mental health specialists, e.g. psychotherapists, psychiatrists, and psychiatric nurses. Labor costs were calculated based on gross hourly wage data from the German official labor cost index [64]. The mean intervention costs per patient were added to the total costs of patients in the CSC group during the 12-month follow-up.

### Data analysis

The main data analysis was conducted according to the intention-to-treat principle. As missing information on the 215 included variables varied between 0.3% and 55.8%, and 37,374 (28.3%) of all 132,225 records were incomplete, missing information was imputed assuming missing at random. Thereby, multiple imputation by chained equations (MICE) with predictive mean matching and a total number of m = 20 imputations was used as imputation method [65].

Sample characteristics at T0 and costs and health effects at T0, T1 and T2 were compared using F-tests based on linear regression or (multinomial) logistic regression, whichever was appropriate. Furthermore, costs and health effects during the 12-month follow-up were compared using two-part models with a logit model in the first part and a generalized linear model with a gamma family and log link function for the second part, and generalized linear models with a gamma family and log link function, whichever was appropriate. Each model was estimated with robust standard errors and adjusted for sex, age, occupational status, main diagnosis, study inclusion before COVID-19 pandemic, number of comorbidities, MCS score, and the respective costs/health effect at T0.

The cost-effectiveness of the CSC model compared with TAU was analyzed using the incremental cost-utility ratio (ICUR). The ICUR was calculated as the ratio of the unadjusted difference in mean total costs and the unadjusted difference in mean QALYs between the CSC group and the TAU group during the 12-month follow-up. The uncertainty of the ICUR was assessed using the cost-effectiveness acceptability curve (CEAC) based on net-benefit regressions [66]. CEACs are a common alternative to confidence intervals around the ICUR and to cost-effectiveness planes for visualizing the distribution of uncertainty around the ICUR [67, 68]. The individual net-benefit was used as dependent variable in a linear regression model with robust standard errors and calculated as the product of the patient’s individual 12-month costs and a WTP threshold per QALY gained minus the patient’s individual QALYs, with WTP thresholds ranging from 0€ to 50,000€. Besides the variable distinguishing between the CSC and TAU group, regressions were adjusted by the same independent variables as those used to compare costs and health effects during the 12-month follow-up. The probabilities of cost-effectiveness based on the net-benefit regressions for each WTP threshold were calculated by *p*-value/2 for negative CSC/TAU group coefficients, and by 1 – *p*-value/2 for positive CSC/TAU group coefficients. Finally, CEACs plotted the probabilities of cost-effectiveness against the WTP thresholds per QALY gained.

Multiple imputation and data analyses were performed using Stata/MP 17.0 (StataCorp, TX, USA). All applicable statistics were tested two-sided with a significance level of 0.05. All *p*-values were considered as descriptive only.

### Additional analyses

First, subgroup analyses were conducted for patients with depressive disorders, anxiety disorders, somatoform disorders, or alcohol-related disorders. Second, analyses were repeated with QALYs calculated using the EQ-VAS and SF-6D index as health effect. Third, response to treatment and remission from symptoms were used as alternative measures of health effects. Fourth, the analyses were conducted from a health care payer’s perspective by excluding indirect costs due to absenteeism and reduced productivity at work from the total costs. Fifth, costs winsorized at the 95th percentile and only mental health-related costs (mental health inpatient care, outpatient mental health specialist services, and psychopharmacological medication) were considered in additional analyses. Last, analyses were repeated both as complete case analysis and based on a dataset in which missing information was imputed by the last observation carried forward method.

## Results

### Sample characteristics

The patients in the CSC (*n* = 307) and TAU group (*n* = 308) were on average aged 38 and 43 years, respectively (Table 1). The majority of patients were female (CSC group: 61%, TAU group: 67%), single or never married, did not have a migration background, graduated from intermediate secondary school or high school, and were employed full-time. Almost all patients had statutory health insurance (CSC group: 97%, TAU group: 93%). Fewer patients in the TAU group were included in the study prior to the COVID-19 pandemic (68% vs. 91%) and fewer patients were included as planned within the framework of the recruitment strategy (59% vs. 96%).

**Table 1:** Comparison of sample characteristics at baseline

Characteristics	CSC group (*n* = 307)	TAU group (*n* = 308)
Age: mean years (SE)	37.71 (0.71)	43.26 (0.85)
Female sex: *n* (%)	188 (61.24)	205 (66.56)
Marital status: *n* (%)		
Single (never married)	186 (60.41)	165 (53.75)
Married	77 (25.20)	94 (30.45)
Separated/divorced/widowed	44 (14.40)	49 (15.80)
Migration background: *n* (%)		
No migration background	211 (68.83)	224 (72.71)
Direct migration background^†^	38 (12.18)	39 (12.52)
Indirect migration background^‡^	58 (18.99)	45 (14.77)
Education: *n* (%)		
Secondary school/no school degree	28 (9.06)	41 (13.25)
Intermediate secondary school	90 (29.19)	95 (30.81)
High school	125 (40.80)	107 (34.61)
University or technical college degree	64 (20.96)	66 (21.33)
Occupational status: *n* (%)		
Employed full-time	172 (56.06)	158 (51.31)
Employed part-time	61 (19.67)	65 (21.23)
Marginally employed	15 (4.92)	5 (1.69)
Not employed	59 (19.35)	79 (25.76)
Statutory health insurance: *n* (%)	298 (96.95)	286 (92.84)
Study inclusion before COVID-19 pandemic: *n* (%)	278 (90.55)	209 (67.86)
Recruitment strategy as planned: *n* (%)	294 (95.77)	182 (59.09)
Main diagnosis: *n* (%)		
Depressive disorder	226 (73.62)	241 (78.25)
Anxiety disorder	63 (20.52)	30 (9.74)
Somatoform disorder	18 (5.86)	29 (9.42)
Alcohol-related disorder	0 (0.00)	8 (2.60)
Number of comorbidities: *n* (%)		
No comorbidities	217 (70.68)	211 (68.51)
1 comorbidity	74 (24.10)	62 (20.13)
2 comorbidities	12 (3.91)	26 (8.44)
3 comorbidities	4 (1.30)	9 (2.92)

*CSC* Collaborative and stepped care, *TAU* Treatment as usual, *SE* Standard error

^†^ Persons with their own migration experience who were born without German citizenship

^‡^Persons without their own migration experience who were born to at least one parent with direct migration background

Of all patients, 21% in the CSC group and 10% in the TAU group were screened with an anxiety disorder, 79% and 74% were screened with a depressive disorder and 9% and 6% with a somatoform disorder, respectively. Only patients in the TAU group were screened with an alcohol-related disorder (3%). Furthermore, the majority of patients in the CSC and TAU groups had no comorbidities (71% and 69%, respectively; Table 1).

### Health effects and costs

At T0, the unadjusted mean EQ-5D-5L index score of the CSC group and the TAU group was 0.81 and 0.83, respectively (Table 2). At T1, the unadjusted mean EQ-5D-5L index score increased in both, the CSC group and TAU group (0.89 and 0.88) and remained almost the same at T2 (0.90 and 0.89). The unadjusted mean EQ-5D-5L index scores between groups were not different at any time point. Furthermore, unadjusted mean EQ-VAS scores (68.63 vs. 70.73) and SF-6D index scores (0.70 vs. 0.72) were not different between the CSC group and the TAU group at T1 and remained almost the same at T2 (71.12 vs. 69.82, and 0.71 in both groups).

**Table 2:** Unadjusted mean intervention costs, 6-month total costs and health effects

Cost category/Measure of health effect	CSC group (*n* = 307)	TAU group (*n* = 308)	Difference CSC-TAU	*P*-value^‡^
Intervention costs: mean (SE)				
T0-T2	139€ (0€)	-	139€ (0€)	
Total costs (PP): mean (SE)				
T0	1,250€ (175€)	1,500€ (1,839€)	−250€ (258€)	0.333
T1	2,146€ (250€)	2,490€ (263€)	−344€ (374€)	0.359
T2	2,484€ (275€)	2,518€ (295€)	−34€ (391€)	0.931
Total costs (SP): mean (SE)				
T0	16,483€ (601€)	13,801€ (643€)	2,682€ (889€)	0.003
T1	14,989€ (758€)	13,055€ (814€)	1,953€ (1,089€)	0.077
T2	12,773€ (1,139€)	11,117€ (1,021€)	1,657€ (1,543€)	0.285
EQ-5D-5L-Index: mean (SE)				
T0	0.81 (0.01)	0.83 (0.01)	−0.02 (0.02)	0.206
T1	0.89 (0.01)	0.88 (0.01)	0.01 (0.02)	0.493
T2	0.90 (0.01)	0.89 (0.01)	0.01 (0.01)	0.605
EQ-VAS: mean (SE)				
T0	63.91 (1.17)	67.68 (1.22)	−3.76 (1.72)	0.030
T1	68.63 (1.40)	70.73 (1.52)	−2.10 (2.0)	0.296
T2	71.12 (1.63)	69.82 (1.62)	1.29 (2.35)	0.584
SF-6D: mean (SE)				
T0	0.59 (0.01)	0.64 (0.01)	−0.04 (0.01)	< 0.001
T1	0.70 (0.01)	0.72 (0.01)	−0.02 (0.01)	0.089
T2	0.71 (0.01)	0.71 (0.01)	0.00 (0.01)	0.938

*T0* baseline, *T1* 6 months after baseline, *T2* 12 months after baseline, *CSC* Collaborative and stepped care, *TAU* Treatment as usual, *PP* Health care payer’s perspective, *SP* Societal perspective, *SE* Standard error

^‡^Based on F-test

There was no difference in adjusted mean QALYs based on the EQ-5D-5L index between the CSC group and the TAU group during the 12-month follow-up period (+0.00; 95% CI −0.03 to 0.03; *p* = 0.897; CSC group 0.86; TAU group 0.86; Table 3). Furthermore, there was no difference in adjusted mean QALYs based on the EQ-VAS (−0.01; 95% CI −0.03 to 0.02; *p* = 0.726) and SF-6D index (−0.01; 95% CI −0.02 to 0.01; *p* = 0.330) between groups.

**Table 3:** Adjusted^¶^ differences between CSC and TAU in mean costs and health effects during 12-month follow-up

Cost category/Measure of health effect	CSC group (*n* = 307)	TAU group (*n* = 308)	Difference CSC-TAU	*P*-value
Inpatient care and rehabilitation: mean (SE)	2,022€ (396€)	2,490€ (409€)	−468€ (611€)	0.443
Outpatient physician services: mean (SE)	2,199€ (115€)	1,542€ (110€)	657€ (182€)	< 0.001
Somatic outpatient physician services: mean (SE)	745€ (52€)	786€ (55€)	−38€ (69€)	0.579
Outpatient mental health services: mean (SE)	1,472€ (101€)	744€ (89€)	685€ (146€)	< 0.001
Outpatient non-physician services: mean (SE)	205€ (24€)	239€ (28€)	−34€ (38€)	0.374
Medical aids: mean (SE)	11€ (2€)	11€ (2€)	0€ (3€)	0.924
Medication: mean (SE)	373€ (76€)	365€ (82€)	8€ (68€)	0.909
Nursing care: mean (SE)	88€ (79€)	150€ (126€)	−62€ (87€)	0.474
Indirect costs: mean (SE)	20,961€ (2,140€)	19,806€ (2,192€)	1,155€ (1,971€)	0.558
Absenteeism: mean (SE)	8,664€ (769€)	6,942€ (819€)	1,722€ (1,186€)	0.147
Presentism: mean (SE)	13,240€ (871€)	13,896€ (1,240€)	−657€ (1,597€)	0.681
Total costs (PP): mean (SE)^&^	5,644€ (1,012€)	5,235€ (998€)	409€ (773€)	0.597
Total costs (SP): mean (SE)^&^	27,174€ (1,706€)	26,441€ (1,792€)	733€ (2,774€)	0.792
QALY (EQ-5D-5L): mean (SE)	0.86 (0.01)	0.86 (0.01)	0.00 (0.01)	0.897
QALY (EQ-VAS): mean (SE)	0.69 (0.01)	0.69 (0.01)	−0.01 (0.01)	0.726
QALY (SF-6D): mean (SE)	0.68 (0.00)	0.69 (0.01)	−0.01 (0.01)	0.330

*PP* health care payer’s perspective, *SP* societal perspective, *SE* Standard error, *QALY* Quality-adjusted life year, *CSC* Collaborative and stepped care, *TAU *Treatment as usual

^¶^ Adjusted for sex, age, occupational status, main diagnosis, study inclusion before COVID-19 pandemic, number of comorbidities, MCS and the respective costs/health effect at baseline by a two-part model/generalized linear model with robust standard errors

^&^ Includes intervention costs

From the societal perspective, the unadjusted mean 6-month total costs were 16,483€ in the CSC group and 13,801€ in the TAU group at T0 (Table 2). At T1 and T2, the unadjusted mean total costs were 14,989€ and 12,773€ in the CSC group, and 13,055€ and 11,117€ in the TAU group, respectively. The unadjusted mean total costs between groups were different only at T0 (+2,682€). There was no difference in adjusted mean total costs between groups during the 12-month follow-up period (+733€; CSC group 27,174€; TAU group 36,441€; Table 3). With respect to the individual cost categories, only the adjusted mean costs for outpatient mental health services were higher in the CSC group (+685€; 95% CI 398€ to 972€; *p* < 0.001).

### Cost-effectiveness

**Fig. 1:**
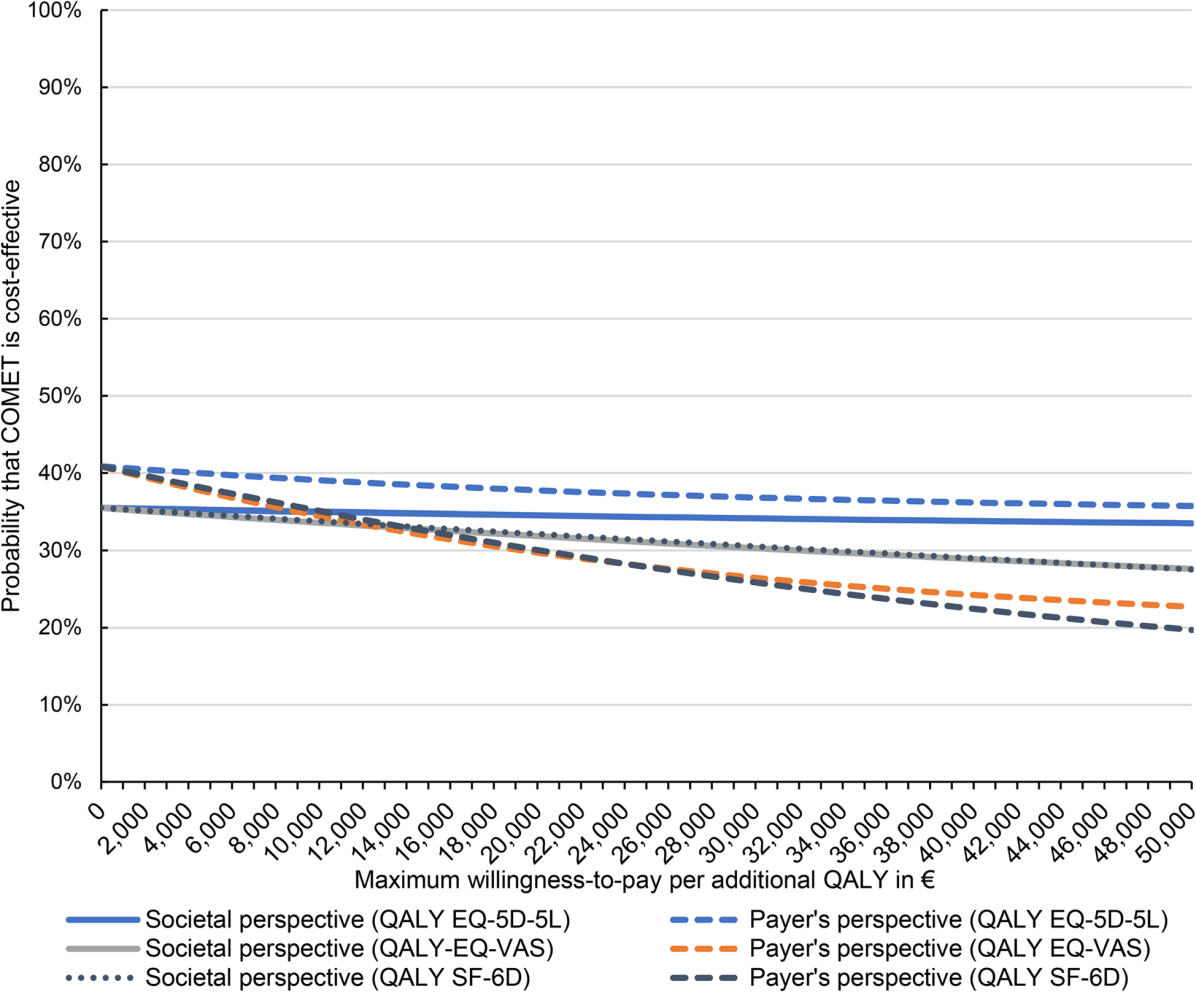
Probability of cost-effectiveness^¶^ of the CSC model compared with TAU – Main analysis^†^ and secondary analyses^‡^ QALY: Quality-adjusted life year, CSC: collaborative and stepped care, TAU: treatment as usual. ^¶^ Adjusted for sex, age, occupational status, main diagnosis, study inclusion before COVID-19 pandemic, number of comorbidities, MCS and the respective total costs/health effect at baseline by a linear regression model with robust standard errors. ^†^Main analysis: societal perspective and QALYs based on the EQ-5D-5L index. ^‡^Secondary analyses: health care payer’s perspective, QALYs based on the EQ-VAS or on the SF-6D index.

The unadjusted ICUR of the CSC model was 1,612,266€ per QALY from the societal perspective, resulting from higher costs (+3,730€) and similar health effects (+0.00 QALY) compared with TAU. The adjusted probability of cost-effectiveness of the CSC model was 35% at a WTP of 0€ per QALY gained (Fig. 1). The adjusted probability of cost-effectiveness decreased to 34% at a WTP of 50,000€ per QALY gained. A cost-effectiveness plane is shown in Figure S1 in Additional file 1.

### Additional analyses

The subgroup analyses for patients with depressive disorders, anxiety disorders or somatoform disorders yielded adjusted probabilities for the cost-effectiveness of the CSC model of 32%, 77% and 43% at a WTP of 0€ per QALY gained, and of 34%, 65% and 13% at a WTP of 50,000€ per QALY gained, respectively (Figure S2 in Additional file 1). A subgroup analysis for patients with alcohol-related disorders was not possible, because no patients from the CSC group were in this subgroup. The adjusted mean QALYs and total costs of the CSC and TAU group during the 12-month follow-up period are shown in Table S1 in Additional file 1.

The additional analysis from a health care payer’s perspective yielded adjusted probabilities of cost-effectiveness for the CSC model of 41% at a WTP of 0€ per QALY gained, and of 36% at a WTP of 50,000€ per QALY gained (Fig. 1). The adjusted mean total costs between CSC and TAU group during the 12-month follow-up period from a health care payer’s perspective were not different (+409€; *p* = 0.597; 95% CI −1107€ to 1925€; CSC group 5,644€; TAU group 5,235€; Table 3). The additional analyses using QALYs as effect measure based on the EQ-VAS and SF-6D index both showed an adjusted probability of cost-effectiveness for the CSC model of 28% at a WTP of 50,000€ per QALY gained, respectively (Fig. 1). There was no difference in adjusted mean QALYs based on the EQ-VAS (−0.01; *p* = 0.726; CSC group 0.69; TAU group 0.69; Table 3) and SF-6D index (−0.01; *p* = 0.330; CSC group 0.69; TAU group 0.69) between the CSC group and the TAU group during the 12-month follow-up period (Table 3).

Additional analyses with response to treatment and remission from symptoms as health effect measures showed adjusted probabilities of cost-effectiveness of the CSC model of 35% at a WTP of 0€ per additional response to treatment/remission from symptoms, and of 79%/61% at a WTP of 50,000€ per additional response to treatment/remission from symptoms (Figure S3 in Additional file 1). The analysis with costs winsorized at the 95th percentile and with only mental health-related costs showed adjusted probabilities of cost-effectiveness of the CSC model of less than 50% at any WTP per QALY gained. Furthermore, both, the complete case analysis and the analysis based on a dataset in which missing information was imputed by the last observation carried forward method showed adjusted probabilities of cost-effectiveness of the CSC model of less than 50% at any WTP per QALY gained (Figure S4 and Table S2 in Additional file 1).

## Discussion

The cost-effectiveness of the CSC model compared with TAU in patients with depressive, anxiety, somatoform or alcohol-related disorders and their comorbidities in German primary care was unlikely from a societal perspective. The low probability of cost-effectiveness below 50% even decreased with higher WTP per QALY gained. The CEAC reflects a joint density of incremental costs and incremental effects, in which 35% involved cost-savings and less than 2% involved QALY gains [67, 68]. Furthermore, despite the relatively low mean intervention costs per patient (139€), the adjusted costs analysis revealed higher costs for outpatient mental health services within the CSC group. This finding indicates a higher utilization of psychotherapists, psychiatrists, and other mental health specialists of patients in the CSC group. Hence, the CSC network might have overcome the hurdle of low cross-disciplinary and coordinated cooperation between GPs and mental health specialists, as well as the effort patients have to make themselves to find a psychotherapist [69, 70]. It must be acknowledged that these higher costs for outpatient mental health services within the CSC group had no impact on the mean total costs between the groups during the 12-month follow-up period, which did not differ. However, this economic evaluation is still meaningful, as it aimed at estimating the probability of the ICER to be acceptable at a given WTP rather than testing the hypotheses that the CSC is more effective and less costly [71].

The low probability of cost-effectiveness of the CSC model compared with TAU was robust to the cost perspective, the variation of the health effect measure used to calculate QALYs, and the use of response to treatment or remission from symptoms as alternative health effect. Furthermore, the result was robust to the choice of the imputation method and to potential cost outliers. Similarly, a low probability of cost-effectiveness of the CSC model compared with TAU was found in subgroup analyses of patients with depressive disorders, anxiety disorders or somatoform disorders.

The EQ-5D-5L index scores, which were used to calculate the QALYs for the ICUR, of patients in both the CSC and TAU group were high already at baseline (0.81 and 0.83), resulting in QALYs of 0.86 during the 12-month follow-up. It is known that generic HrQoL measures, such as the EQ-5D-5L, may not be sufficiently sensitive to capture the recovery process of depressive, anxiety, somatoform or alcohol-related disorders in the context of mental health services [72]. Among patients with, among others, depressive, anxiety or somatoform disorders the QALYs were 0.73 and 0.71 during the 12-month follow-up in a randomized controlled trial which compared stepped integrated care with TAU in Germany [73]. This trial, however, also included patients with more severe mental disorders that are likely to have a greater impact on the EQ-5D-5L, such as schizophrenia spectrum disorder or personality disorder. Compared with a representative German population sample, the mean EQ-5D-5L index scores at baseline of the patients in the current study were still lower than the population average (0.88) [74].Cost-effectiveness analyses of other CSC models in Germany conducted in the past showed similar results for patients with depressive disorders and somatoform disorders for the most part: For patients with depressive disorders, the probability of cost-effectiveness of a four-level stepped care model was below 10% at a WTP of 50,000€ per additional QALY [4]. However, for older patients with depressive disorders, CSC models were cost-effective with a probability of 80% at a WTP of 70€ per additional depression-free day [7]. The probability of cost-effectiveness of a CSC model for patients with somatoform and functional disorders was only 31% at a WTP of 10,000€ per additional response to treatment [5]. A nurse-led CSC model for patients with anxiety, depressive or somatic symptoms was only cost-effective with a probability of 49% at a WTP of 50,000€ per additional QALY [6].

To the best of our knowledge, the cost-effectiveness of CSC models for patients with depressive, anxiety, somatoform or alcohol-related disorders and their comorbidities has not yet been evaluated in a German setting. Only the cost-effectiveness of a primary care practice team-delivered exposure training for patients with panic disorder has been evaluated [75]. The probability of cost-effectiveness was 96% at a WTP of 50,000€ per additional QALY. For the United Kingdom, the cost-effectiveness of a stepped care intervention for older hazardous alcohol users in primary care was evaluated [21]. The probability of cost-effectiveness of this intervention was 94% at a WTP of 30,000£ (about 35,300€) per additional QALY.

All the studies mentioned above analyzing the cost-effectiveness of CSC models in Germany [4–7] were conducted in the metropolitan region Hamburg. There is some tendency that CSC models focusing on depressive and somatoform disorders that are implemented in the existing structures of primary care practices are not likely to be cost-effective. Yet, there is evidence that CSC models might be a cost-effective alternative to TAU for certain groups of patients with mental disorders (e.g., patients with moderate depression or somatoform symptoms, older patients) [4, 5, 7]. In general, it was repeatedly noted that cost-effectiveness analyses of CSC models have weaknesses with respect to the choice of health effect measure used to calculate QALYs, the time horizon, the sample size, the cluster randomization at the primary care practice level and not least, the existing complex structures of outpatient physician services in which the models were implemented [4–7]. Despite all that, GPs positively evaluated the current CSC model with respect to its ability to reduce waiting times for patients, and to increase personal contacts and mutual knowledge [43]. Yet, it remains unclear whether the promotion of those specific characteristics of CSC models would lead to a cost-effective care of patients with mental disorders in the outpatient setting.

### Strenghts and limitations

In the COMET study, a CSC model for patients with the most common mental disorders was successfully developed and implemented in the existing structures of primary care practices in Germany without resorting to the support of additional care managers. Furthermore, this study was the first to evaluate the cost-effectiveness of a comprehensive CSC model for four of the most common mental disorders. The data analyses were based on advanced statistical methods, such as MICE with predictive mean matching as imputation method and the assessment of the uncertainty of the ICUR using a CEAC based on covariate-adjusted net-benefit regressions.

There are also important limitations that have to be mentioned. First, the sample characteristics at baseline differed between the CSC and TAU group. Furthermore, the mean total costs at baseline were significantly different in patients of the CSC and TAU group (+2,682€), whereas the mean total costs did neither differ significantly at T1 (+1,953€) nor at T2 (+1,657€). However, even after adjusting for these baseline differences, the adjusted 12-month total costs from a societal perspective did not differ significantly between the CSC and TAU group. Yet, it remains uncertain whether other unobserved differences between the CSC and TAU group might have had an impact on response to treatment as well as health care utilization. Second, the recruitment of patients within primary care practices may have led to a selection bias. Primary care practices of the TAU group had difficulty in recruiting patients in time. In order to achieve the required sample size, the recruitment strategy had to be changed during the ongoing study [14, 42]. In those primary care practices with difficulty recruiting patients, one person from the study team supported recruitment. Furthermore, the onset of the COVID-19 pandemic was during the patient recruitment period, which might have led to a further selection bias, as patients of the TAU group were recruited later. However, the change in recruitment strategy did not affect randomization at the primary care practice level and the results were robust to the adjustment for inclusion of patients to the study before or after the onset of the COVID-19 pandemic. Third, loss to follow-up as well as missing information across variables was high. However, MICE with predictive mean matching was used as imputation method. By multiple imputation, statistical inferences are to be considered valid even for higher proportions of missing data, if the underlying missing data mechanisms were (completely) at random [76, 77]. Furthermore, additional analyses showed that the study results were robust to alternative ways of dealing with missing values, such as using the last observation carried forward method or using complete cases in the analysis only. Fourth, the CSC model may not have been fully implemented as intended, as it may have been overly complex for patients with multiple disorders and for the network of health care providers. Although patients in the CSC group had a higher utilization of psychotherapists, psychiatrists, and other mental health specialists, this does not necessarily reflect quality or appropriateness of treatment. Fifth, the primary endpoint of the corresponding effectiveness analysis of the CSC model mental HrQoL at 6 months after baseline, whereas this cost-effectiveness analysis used the 12-month follow-up. Thus, comparability of the effectiveness and cost-effectiveness results with regard to the length of follow-up is limited. For economic evaluations, however, it would be ideal to consider the costs and health effects over a long period of time [78].

### Conclusion

The CSC model was not likely to be cost-effective from a societal perspective compared with TAU in patients with depressive, anxiety, somatoform or alcohol-related disorders and their comorbidities in German primary care. However, the GPs of the CSC group were able to refer patients more frequently to psychotherapists and psychiatrists through the participating network of health care providers. Further research is needed to identify the elements of the CSC model that have an influence on cost-effectiveness and to analyze the cost-effectiveness with a long-term follow-up.

## Supplementary Information

Additional file 1. Tables S1-S2 and Figures S1-S4. Table S1: Adjusted differences between CSC group and TAU group in mean total costs and health effects by main diagnosis during 12-month follow up (*n* = 607) – Subgroup analyses. Table S2: Adjusted differences between the CSC group and TAU group in mean total costs and health effects during 12-month follow up – Additional analyses. Figure S2: Probability of cost-effectiveness of the CSC model compared with TAU for different thresholds for maximum willingness-to-pay per additional QALY – Subgroup analyses (Persons with affective disorders, persons with anxiety disorders, persons with somatoform disorders). Figure S1: Adjusted cost-effectiveness plane of the CSC model compared with TAU – Analysis from societal perspective. Figure S2: Probability of cost-effectiveness of the CSC model comared with TAU for different thresholds for maximum willingness-to-pay per additional QALY – Secondary analyses (Analyses with response to treatment and remission from symptoms as measure of health effect). Figure S2: Probability of cost-effectiveness of the CSC model compared with TAU for different thresholds for maximum willingness-to-pay per additional QALY – Sensitivity analyses (Analysis with winsorized costs, analysis with last observation carried forward, complete case analysis, analysis with psychiatric costs).

## Data Availability

The data that support the findings of this study are available on reasonable request from the corresponding author (TG). The data are not readily available due to missing permission from participants to share anonymized participant data publicly. The analytic code and research materials associated with this manuscript are available on reasonable request from the corresponding author (TG), only for purposes of reproducing results or replicating procedures.

## Abbreviations

AUDIT: Alcohol Use Disorders Identification Test
CEAC: Cost-effectiveness acceptability curve
CSC: Collaborative and stepped care
EQ-5D-5L: Five-level version of the EQ-5D
EQ-VAS: Visual analogue scale of the EQ-5D-5L
GAD-7: Patient Health Questionnaire module to screen for generalized anxiety disorder
GP: General practitioner
HrQoL: Health-related quality of life
ICUR: Incremental cost-utility ratio
MCS: Mental component summary
MICE: Multiple imputation by chained equations
PHQ-15: Patient Health Questionnaire module to screen for somatoform disorders
PHQ-9: Patient Health Questionnaire module to screen for major depressive disorder
PP: Health care payer’s perspective
SE: Standard error
SP: Societal perspective
TAU: Treatment as usual
QALY: Quality-adjusted life year
WTP: Willingness-to-pay
