# Supplementary material for: Cost-utility analysis of a collaborative and stepped care model in patients with mental disorders in German primary care (the COMET study)
Source: BMC Psychiatry. 2025 Oct 13;25:973. doi: 10.1186/s12888-025-07428-5 (PMC12516892; doi:10.1186/s12888-025-07428-5)
Supplement: Supplementary file 1 — Additional file 1. Tables S1-S2 and Figures S1-S4. Table S1: Adjusted differences between CSC group and TAU group in mean total costs and health effects by main diagnosis during 12-month follow up (n = 607) – Subgroup analyses. Table S2: Adjusted differences between the CSC group and TAU group in mean total costs and health effects during 12-month follow up – Additional analyses. Figure S2: Probability of cost-effectiveness of the CSC model compared with TAU for different thresholds for maximum willingness-to-pay per additional QALY – Subgroup analyses (Persons with affective disorders, persons with anxiety disorders, persons with somatoform disorders). Figure S1: Adjusted cost-effectiveness plane of the CSC model compared with TAU – Analysis from societal perspective. Figure S2: Probability of cost-effectiveness of the CSC model comared with TAU for different thresholds for maximum willingness-to-pay per additional QALY – Secondary analyses (Analyses with response to treatment and remission from symptoms as measure of health effect). Figure S2: Probability of cost-effectiveness of the CSC model compared with TAU for different thresholds for maximum willingness-to-pay per additional QALY – Sensitivity analyses (Analysis with winsorized costs, analysis with last observation carried forward, complete case analysis, analysis with psychiatric costs). [file 12888_2025_7428_MOESM1_ESM.docx]

Table S1: Adjusted^¶^ differences between CSC group and TAU group in mean total costs and health effects by main diagnosis^†^ during 12-month follow up (n=607) – Subgroup analyses

| **Cost category / Measure of health effect** | | **CSC group** | **TAU group** | **Difference CSC-TAU** | ***P*-value** |
| --- | --- | --- | --- | --- | --- |
| F30-F39; n=467 | Total costs (PP): mean (SE) | 5874€ (1286€) | 5467€ (1254€) | 407€ (899€) | 0.651 |
|  | Total costs (SP): mean (SE) | 28,593€ (2170€) | 27,243€ (2103€) | 1350€ (3459€) | 0.669 |
|  | QALY (EQ-5D): mean (SE) | 0.86 (0.01) | 0.86 (0.12) | 0.00 (0.02) | 0.786 |
|  | QALY (EQ-VAS): mean (SE) | 0.68 (0.01) | 0.69 (0.01) | −0.01 (0.01) | 0.521 |
|  | QALY (SF-6D): mean (SE) | 0.68 (0.01) | 0.68 (0.01) | −0.01 (0.01) | 0.293 |
| F40, F41; n=93 | Total costs (PP): mean (SE) | 4763€ (938€) | 4144€ (1411€) | 619€ (1699€) | 0.716 |
|  | Total costs (SP): mean (SE) | 23,121€ (2380€) | 27,066€ (6726€) | −3945€ (7806€) | 0.613 |
|  | QALY (EQ-5D): mean (SE) | 0.89 (0.01) | 0.89 (0.05) | −0.01 (0.03) | 0.765 |
|  | QALY (EQ-VAS): mean (SE) | 0.71 (0.02) | 0.72 (0.04) | −0.01 (0.05) | 0.840 |
|  | QALY (SF-6D): mean (SE) | 0.71 (0.01) | 0.71 (0.02) | −0.00 (0.02) | 0.874 |
| F45; n=47 | Total costs (PP): mean (SE) | 4935€ (2248€) | 4532€ (1911€) | 403€ (3253€) | 0.901 |
|  | Total costs (SP): mean (SE) | 25,147€ (7306€) | 24,599€ (6776€) | 548€ (9624€) | 0.955 |
|  | QALY (EQ-5D): mean (SE) | 0.79 (0.05) | 0.86 (0.06) | −0.07 (0.09) | 0.431 |
|  | QALY (EQ-VAS): mean (SE) | 0.68 (0.04) | 0.71 (0.03) | −0.02 (0.05) | 0.647 |
|  | QALY (SF-6D): mean (SE) | 0.69 (0.03) | 0.71 (0.02) | −0.02 (0.04) | 0.592 |

F30-F39: mood disorders, F40: phobic anxiety disorders, F41: other anxiety disorders, F45: somatoform disorders, PP: payer’s perspective, SP: societal perspective, SE: Standard error, QALY: Quality-adjusted life year, CSC: collaborative and stepped care model, TAU: treatment as usual, VAS: visual analogue scale

^¶^ Adjusted for sex, age, occupational status, main diagnosis, study inclusion before COVID-19 pandemic, number of comorbidities, MCS and the respective costs / health effect at baseline by a two-part model/generalized linear model with robust standard errors

^†^ F10: Mental and behavioral disorders due to use of alcohol was omitted because this diagnosis was not present in the CSC group

Table S2: Adjusted^¶^ differences between the CSC group and TAU group in mean total costs and health effects during 12-month follow up – Additional analyses

| **Cost category / Measure of health effect** | | **N** | **CSC group** | **TAU group** | **Difference CSC-TAU** | ***P*-value** |
| --- | --- | --- | --- | --- | --- | --- |
| Complete case analysis | Total costs (PP): mean (SE) | 170 | 4423€ (455€) | 4743€ (734€) | −320€ (937€) | 0.732 |
|  | Total costs (SP): mean (SE) | 148 | 23,281€ (1722€) | 23,336€ (2173€) | −55€ (3272€) | 0.987 |
|  | QALY (EQ-5D-5L): mean (SE) | 253 | 0.86 (0.01) | 0.87 (0.01) | −0.01 (0.02) | 0.725 |
|  | QALY (EQ-VAS): mean (SE) | 243 | 0.69 (0.01) | 0.71 (0.01) | −0.02 (0.02) | 0.265 |
|  | QALY (SF-6D): mean (SE) | 231 | 0.68 (0.01) | 0.70 (0.01) | −0.01 (0.01) | 0.226 |
| Analysis with LOCF | Total costs (PP): mean (SE) | 357 | 4529€ (659€) | 3838€ (689€) | 691€ (644€) | 0.284 |
|  | Total costs (SP): mean (SE) | 353 | 29361 (1449€) | 26,767€ (1350€) | 2594€ (2257€) | 0.250 |
|  | QALY (EQ-5D-5L): mean (SE) | 446 | 0.84 (0.01) | 0.84 (0.01) | −0.00 (0.01) | 0.839 |
|  | QALY (EQ-VAS): mean (SE) | 442 | 0.69 (0.01) | 0.69 (0.01) | −0.01 (0.01) | 0.511 |
|  | QALY (SF-6D): mean (SE) | 460 | 0.66 (0.00) | 0.66 (0.00) | −0.00 (0.00) | 0.560 |
| Analysis with only mental health costs | Total costs: mean (SE) | 615 | 2219€ (193€) | 1424€ (166€) | 795€ (225€) | < 0.001 |
| Analysis with winsorized costs^†^ | Total costs (PP): mean (SE) | 615 | 4811€ (336€) | 4171€ (347€) | 640€ (498€) | 0.199 |
|  | Total costs (SP): mean (SE) | 615 | 25,845€ (1315€) | 24.813€ (1487€) | 1032€ (2280€) | 0.651 |

PP: payer’s perspective, SP: societal perspective, SE: Standard error, QALY: Quality-adjusted life year, CSC: collaborative and stepped care model, TAU: treatment as usual

^¶^ Adjusted for sex, age, occupational status, main diagnosis, study inclusion before COVID-19 pandemic, number of comorbidities, MCS and the respective costs / health effect at baseline by a two-part model/generalized linear model with robust standard errors

^†^ winsorized at the 95^th^ percentile


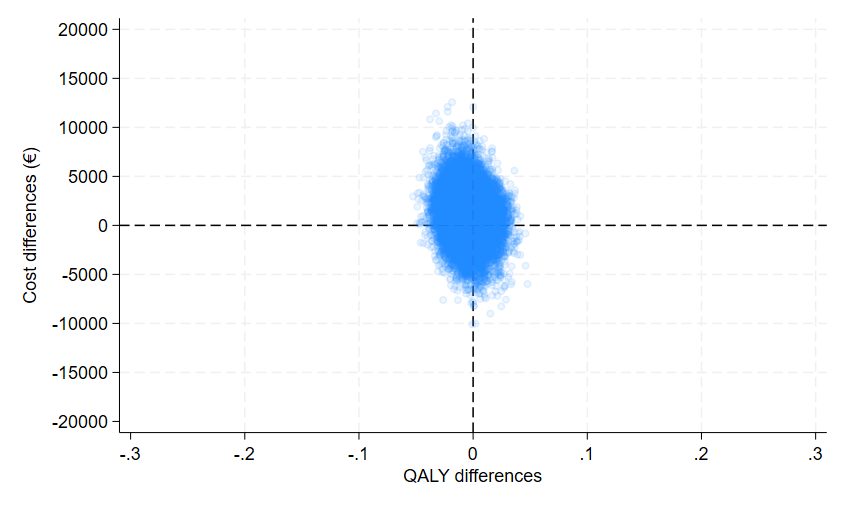


**Figure S1: Adjusted^¶^ cost-effectiveness plane of the CSC model compared with TAU – Analysis from societal perspective^†^**

CSC: collaborative and stepped care model, TAU: treatment as usual, QALY: Quality-adjusted life year

^¶^ Adjusted for sex, age, occupational status, main diagnosis, study inclusion before COVID-19 pandemic, number of comorbidities, MCS and the EQ-5D-5L index at baseline / total costs up to 6 months before baseline by a seemingly unrelated regression model with bootstrapped standard errors; ^†^ QALYs based on the EQ-5D-5L index

**Figure S2: Probability of cost-effectiveness**^¶^ **of the CSC model compared with TAU for different thresholds for maximum willingness-to-pay per additional QALY – Subgroup analyses (Persons with affective disorders, persons with anxiety disorders, persons with somatoform disorders)**

QALY: Quality-adjusted life year

^¶^ Adjusted for sex, age, occupational status, main diagnosis, study inclusion before COVID-19 pandemic, number of comorbidities, MCS and the respective costs / health effect at baseline by a linear regression model with robust standard errors

**Figure S3: Probability of cost-effectiveness**^¶^ **of the CSC model compared with TAU for different thresholds for maximum willingness-to-pay per additional QALY – Secondary analyses (Analyses with response to treatment and remission from symptoms as measure of health effect)**

QALY: Quality-adjusted life year

^¶^ Adjusted for sex, age, occupational status, main diagnosis, study inclusion before COVID-19 pandemic, number of comorbidities, MCS and the respective costs / health effect at baseline by a linear regression model with robust standard errors

**Figure S4: Probability of cost-effectiveness**^¶^ **of the CSC model compared with TAU for different thresholds for maximum willingness-to-pay per additional QALY – Sensitivity analyses (Analysis with winsorized costs, analysis with last observation carried forward, complete case analysis, analysis with psychiatric costs)**

QALY: Quality-adjusted life year, LOCF: last observation carried forward

^¶^ Adjusted for sex, age, occupational status, main diagnosis, study inclusion before COVID-19 pandemic, number of comorbidities, MCS and the respective costs / health effect at baseline by a linear regression model with robust standard errors
